# The Role of Visual Experience in Auditory Space Perception around the Legs

**DOI:** 10.1038/s41598-019-47410-2

**Published:** 2019-07-29

**Authors:** Elena Aggius-Vella, Claudio Campus, Andrew Joseph Kolarik, Monica Gori

**Affiliations:** 1 U-VIP: Unit for Visually Impaired people, Center for Human Technologies, Istituto Italiano di Tecnologia, Genoa, Italy; 2 DIBRIS Department, University of Genoa, Genoa, Italy; 3 Department of Psychology, University of Cambridge, Downing Street, Cambridge, CB2 3EB UK; 4 Vision and Eye Research Institute, School of Medicine, Anglia Ruskin University, YST 213, Young Street, Cambridge, CB1 2LZ UK; 5 Centre for the Study of the Senses, Institute of Philosophy, University of London, Senate House, Malet Street, London, WC1E 7HU UK

**Keywords:** Auditory system, Cognitive neuroscience

## Abstract

It is widely accepted that vision plays a key role in the development of spatial skills of the other senses. Recent works have shown that blindness is often associated with auditory spatial deficits. The majority of previous studies have focused on understanding the representation of the upper frontal body space where vision and actions have a central role in mapping the space, however less research has investigated the back space and the space around the legs. Here we investigate space perception around the legs and the role of previous visual experience, by studying sighted and blind participants in an audio localization task (front-back discrimination). Participants judged if a sound was delivered in their frontal or back space. The results showed that blindfolded sighted participants were more accurate than blind participants in the frontal space. However, both groups were similarly accurate when auditory information was delivered in the back space. Blind individuals performed the task with similar accuracy for sounds delivered in the frontal and back space, while sighted people performed better in the frontal space. These results suggest that visual experience influences auditory spatial representations around the legs. Moreover, these results suggest that hearing and vision play different roles in different spaces.

## Introduction

How we develop a spatial representation of the environment is a topic that has been widely studied over the last decades^1–4^. There is a general consensus about the crucial role of visual experience in guiding the maturation of spatial cognition^5–7^. Vision has advantages over the other senses in encoding spatial information because it ensures the simultaneous perception of multiple stimuli in the environment^8,9^. This idea is supported by recent studies which showed that vision is important for auditory spatial calibration^4,10–12^. However, few studies^13–18^ have investigated the perception of space around different parts of the body, and the role of vision in calibrating auditory space in areas other than space that is frontal relative to the individual. The current study investigated the effect of blindness in auditory front-back discriminations around the legs above and below the knee, and the influence of visual information in auditory representations of frontal and back space.

The extent of the influence of visual calibration on the auditory domain and the role of visual experience in the development of auditory space perception is still an open issue. For example, not all space around the body can be reached by vision. While vision allows accurate mapping of the frontal space, especially around the upper body part, vision is not available in the back. Blindness is a condition that offers the possibility to study the role of vision on the development of auditory spatial perception. The lack of vision leads to changes in the perception and elaboration of sounds at a neural level^19,20^. An expansion in areas responsive to auditory stimuli and a decrement in signal response latencies was reported for blind individuals^21^. Early blind individuals showed more efficient processing of auditory stimuli^22^. However, the nature of these modifications is still unclear. A review^23^ shows contrasting results on the relation between lack of vision and spatial representation. While some studies have shown enhancements in some aspects of spatial representation, others have reported an impairment in some features of spatial hearing. For example, it has been shown that blind individuals have improved skills for azimuthal localization^24–29^ and relative distance discrimination^30–32^. The recruitment of occipital areas, deprived of their normal visual inputs, have been reported to be responsible for enhanced performance^33,34^. On the other hand, other works have shown that some spatial skills are impaired in the absence of visual input, as shown in early-blind humans such as the localization of the end point of a dynamic sound^11^, the building of an audio metric spatial representation^4^, the evaluation of absolute distance^30^, the auditory spatial representations of the extrapersonal space with different sounds^35^, and the vertical localization of a sound source^36^.

These studies have concentrated their efforts in frontal space, where vision likely plays a principal role in calibrating hearing^37–39^. Although fewer studies have compared auditory spatial perception in frontal and back space^17,40^, results have shown that blind and sighted people are similarly accurate in localizing sound in the frontal space, while in the back and median space, blind people outperform sighted^31,41,42^. However, these studies investigated space around the head, where stimuli have a greater saliency compared to other parts of the body^43^. Only a few studies that we are aware of have investigated the spatial representations of other spaces around the body in sighted people, showing the existence of different peripersonal space representations, not fully independent from each other, but with the trunk as common reference frame^13,44^. Evidence that different spaces are not similarly processed by the brain was found by Aggius-Vella *et al*.^17^, who showed different performance in localizing frontal and back sounds by sighted individuals. While the visual system is highly accurate in spatial localization around the eye level^45^, it is still not clear if and how vision influences space around the lower part of the body.

In this work, we investigated auditory perception and the role of prior visual experience in the space around the legs. Auditory sound localization for front-back discriminations was measured in spaces where it is naturally possible to see (frontal space) and where it is not (back space), considering the body region between the waist and the foot. This task is particularly difficult, as ambiguity in binaural timing (interaural time difference, ITD) and level (interaural level difference, ILD) information often causes front-back confusions in sound localization. In order to understand the role of visual sensory experience in auditory perception in these spaces, auditory spatial localization was measured in sighted and, for the first time, in blind participants. Previous evidence^17,46,47^ suggests that the senses can differently influence or shape sensory information delivered in different areas of space. Studies have shown that blindness results in enhanced auditory localization performance for areas other than frontal relative to the participant, such as for azimuth judgements in peripheral space^26,31,39^. If vision is important for discriminating frontal from back sounds, we should find no differences between front and back space in blind people. Otherwise, it may be possible that blind people perform better in a front-back discrimination task in back space compared to the front^48^. This study investigated for the first time auditory perception in the space around the legs. We investigated this space for its particular features that could lead to different representations between sighted and blind participants. While walking, movement (that it is known to influence spatial representation^49^) produces sound and tactile feedback, both anchored to the feet in the lower part of the leg’s space. This particularity could help blind people in recalibrating spatial representation. However, the general hypothesis (of which this paper is just a part) is that if different neural mechanisms are involved to code different spatial areas, we should find that this kind of audio-tactile recalibration is more evident for space where natural visual calibration is not possible (i.e. the back) and where the audio tactile feedback happens (around the feet). To test this last point, we split the space around the legs into two segments: above and below the knee. The knee divides the leg into two separate segments, allowing free movement. Moreover, the two separate segments are involved in different ways in walking and receiving different sensory feedback. The area below the knee is mostly represented by tactile/proprioceptive and auditory feedback produced by walking, while the area above the knee drives leg movement and receives proprioceptive feedback, but it does not produce any sound. However, in the frontal space around the upper part of the legs, hands are frequently used and their movement is guided by vision, thus vision is likely the predominant sense in this space. In the frontal space, we expected better performance of the sighted compared with the blind group, as previous work found that vision calibrates hearing in spatial bisection tasks^44^. Moreover, it is possible that blind people perform similarly in the front and back space. We expected no differences between groups for space above and below the knee. These two spaces, indeed, can be well calibrated by sensorimotor feedback^47^.

## Method

### Subjects

Twenty-four participants were tested, N = 10 blind subjects (5 females, mean age and standard deviation 31.8 ± 5.4 years old, height: 167 ± 9 cm, see Table 1) and N = 14 blindfolded sighted subjects (7 females, mean age and standard deviation 27.5 ± 5.9 years old, height 169 ± 8 cm). A between-subjects t-test confirmed that the groups of blind and sighted subjects were age matched, t _(22)_ = 1.144, P = 0.2. Participants were recruited on the basis of similar duration and level of school education (high school degree), normal hearing (assessed by an audiometric test), they reported themselves to have no cognitive impairments and to be right handed. The participants provided written informed consent in accordance with the Declaration of Helsinki, and the study was approved by the ethics committee of the local health service (Comitato Etico, ASL3 Genovese, Italy).

**Table 1:** Clinical details of blind participants.

Subjects	Pathology	Age Complete Blindness	Residual Vision at Test	Blindness Duration (yrs)
Subj. 1	Retinopathy of Prematurity	Before birth	No vision	50
Subj. 2	Congenital glaucoma	Before birth	No vision	57
Subj. 3	Retinopathy of Prematurity	Before birth	No vision	29
Subj. 4	Corneal opacity	At 17 years of age, visual performance decreased	No vision	11
Subj. 5	Retinitis pigmentosa	Residual vision started at 10 yr	Lights and shadows	Residual
Subj. 6	Leber’s amaurosis	At 46 years of age	No vision	5
Subj. 7	Retinopathy of Prematurity	Before birth	No vision	51
Subj. 8	Maculopathy	Residual vision started at 69 yr	Residual vision	Residual
Subj. 9	Uveitis	Before birth	Lights and shadows	Residual
Subj. 10	Congenital cataracts	Before birth	No vision	34

The table shows the pathology (self-reported by the participants and based on a certification of blindness provided by a medical doctor), the visual status of each participant and the blindness duration.

### Set up

**Figure 1:**
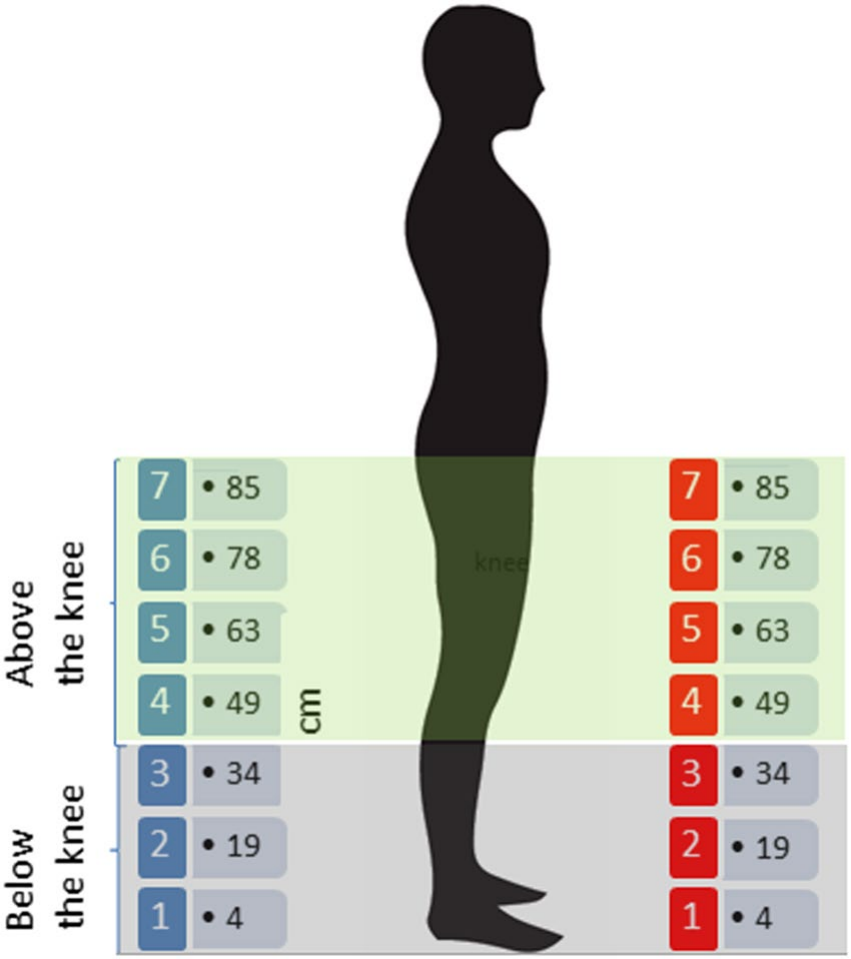
The sound localization task. 14 speakers were split into two arrays of 7 speakers each, vertically oriented. Speakers of each array were positioned at 4, 19, 34, 49, 63, 78 and 85 cm from the floor, leading to 7 equivalent speakers vertical positions in the frontal and back space. The two arrays were positioned facing each other.

The experiment was performed in a dimly lit room. As shown in Fig. 1, the apparatus consisted of 14 speakers split into two identical arrays of 7 speakers each (red and blue squares), vertically oriented. The lower speaker of each array was positioned at 4 cm from the floor, the others were situated at 19, 34, 49, 63, 78 cm and the highest was at 85 cm, leading to 7 equivalent speaker vertical positions (i.e. 7 in the frontal space, and 7 in the back space).

The two arrays were positioned facing each other. One array of speakers was placed in the frontal space (red array), slightly to the left (at 40° in relation to the face) and the other one in the back space (blue array), slightly to the left (at 160° in relation to the face). This configuration was adopted to reduce front-back error but maintaining task difficulty. The different angle size with respect to the midline (40° in the frontal space and −20° in the back space) made the frontal and rearward ITD and ILD values slightly different, helping the participant to discriminate between frontal and rearward sounds. Both arrays were situated at a distance of 50 cm from the position of the participant.

During each trial, pink noise lasting 1 second was randomly delivered from one of the 14 speakers. Each speaker delivered the sound in six trials, for a total of 84 trials (42 trials in the frontal space and 42 in the back space). In order to clarify the representation of low space, for the analysis, we split the 7 equivalent speakers into two areas: 1) space above the knee (speaker numbers 4, 5, 6, 7, above 34 cm), and 2) space below the knee (speakers 1, 2, 3, under 34 cm). We decided to have the maximum height at knee level following previous work^47^, as this is the main joint in the leg that allows free movements of the two segments.

### Sound localization task

All participants were blindfolded, led into the experimental room, and they then remained standing for the entire session. They were asked to keep their head straight and not to direct it toward the sound. They were instructed to verbally report whether sounds were delivered in the frontal or in the back area, without considering their elevations. Subject position and posture was continuously monitored and, if necessary, corrected by the experimenter. Sounds were administered by a custom made code in Matlab (R2013a, The Math Works, USA). The new trial started after the subject’s answer, without any time restriction. The task took approximately 45 minutes.

### Data analysis and statistics

Localization data were post-processed and analysed by a custom made program in R^50^. The seven sound sources were grouped into two spatial levels: space below the knee (speaker numbers 1, 2, and 3), and above the knee (speaker numbers 4, 5, 6, 7). In order to evaluate the relation between sound localization and the role of senses in representing spaces, we analysed the pool of single trials using generalized linear mixed models (GLMM). In this way, we could estimate the variability of fixed and random effects^51^.

As our independent variable was binomial (1, 0), we applied GLMM with a logit link function and a binomial distribution. The benefit of using the GLMM is that the model takes into account the intrinsic binomial nature of the response variable, and overcomes possible issues with ANOVA related to departures from normality for analysing percentage data. With this analysis, two models were fitted for all subjects, taking into account the individual variability and each vertical speakers (1 to 7) in the responses. We fitted the models to the choices from the localization task using the lme4 package^52^ in the R statistical language^50^. In the first model we took into account the correct answer; we regressed, in each trial, the answers of each subject (1 = correct, 0 = incorrect), as a function of speaker vertical position (above the knee vs below the knee) and longitudinal position (front vs back space) as factors within subjects, while considering group (blind vs sighted) as a between-subjects factor. While, in the second model (same dataset) we considered the perceived location of the sound (1 = frontal, 0 = back), as a function of speaker vertical position (above the knee vs below the knee) and longitudinal position (front vs back space) as factors within-subjects, while group (blind vs sighted) as a factor between-subjects. These factors are included in our model as fixed effects, while subject and the 7 speaker positions were included as random effects. We calculated Analysis of Deviance Tables (using Type II Wald Chi-Square tests) for the models using the car package^53^. For significant effects, we performed post hoc comparisons using the lsmeans package^54^, which computes and contrasts least-squares means (predicted marginal means). We adopted Tukey P adjustment, which uses a multivariate t distribution. Contrasts, with P < 0.05 were considered as significant (corrected P values are reported).

In a second analysis we transformed our dependent variable in terms of percentage. For every subject and every portion of space we calculated the percentage of (1) correct and (2) “front” answers, by dividing the sum of correct or “frontal” answers for the number of trials and multiplied it by 100.

## Results

### Analysis using generalized mixed model

Before starting the analysis, we checked that perception of speaker positions (1, 2, 3, 4, 5, 6, 7) were similar inside speaker vertical position (above/below the knee). We split the dataset into two sub datasets: one related to speaker position above the knee and the other below the knee. On each dataset, we regressed, in each trial, the answers of each subject (1 = correct, 0 = incorrect), as a function of longitudinal position (front vs back space) as factors within subjects, while considering group (blind vs sighted) as a between-subjects factor. No significant interactions were found for speakers above the knee: longitudinal position (P = 0.6), group (P = 0.9) and group and longitudinal position (P = 0.8). The same analysis performed for data below the knee showed no interaction between speaker position and longitudinal position (P = 0.1), group (P = 0.8) and group and longitudinal position (P = 0.8).

#### Analysis of correct answers

Analysis of deviance on correct answer showed a main effect of longitudinal position (X^2^_(1)_ = 7.42, P = 0.006). Accuracy was higher in the frontal than in the back space ((OR) = 2.1+/−0.61, z ratio = 2.55, P = 0.01).

**Figure 2:**
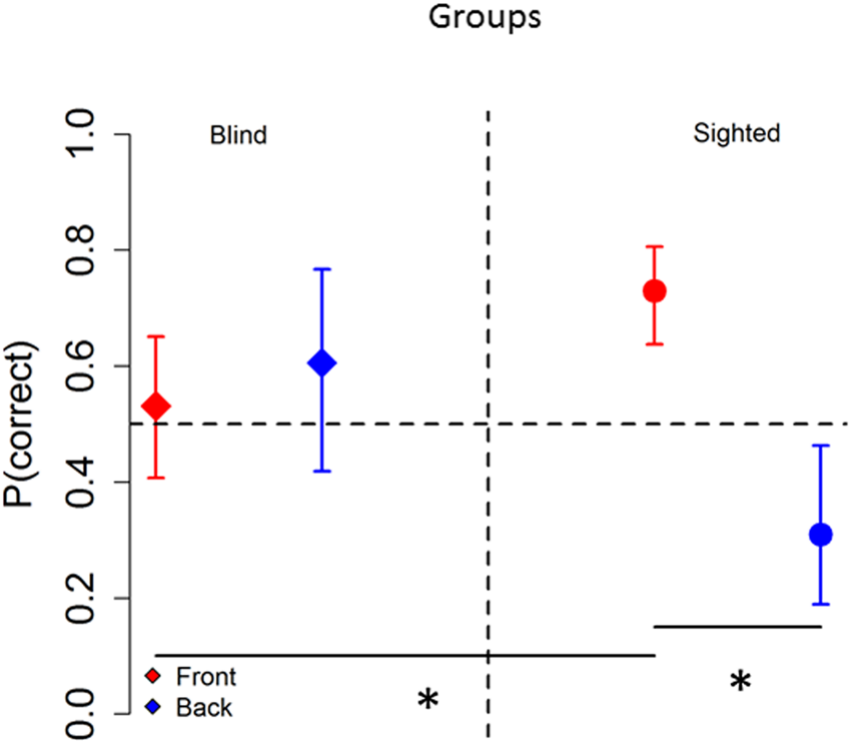
Influence of visual experience: Interaction between group and longitudinal plane. Sighted subjects reported a significantly higher probability to correctly locate sounds delivered in the frontal space (red circle) than in the back space (blue circle), while blind subjects showed no significant differences in localizing sounds presented in frontal and back space. In the frontal space, sighted people performed better than blind people, while no significant differences between groups were found in the back space (horizontal black bars represent significant differences). Symbols represent LS mean, while errors bars indicate the 95% confidence interval estimated by the LS means.

Figure 2 shows the interaction between longitudinal position and group (X^2^_(1)_ = 13.23, P < 0.001). Comparing longitudinal spaces within groups, blind subjects (on the left of Fig. 2) reported a similar probability (0.53 vs 0.60) to correctly locate sounds delivered in the frontal space (red) than in the back space (blue), ((OR) = 0.73+/−0.32, z ratio = −0.69, P = 0.9). Instead, sighted participants (on the right of Fig. 2) showed a higher probability to be correct when sounds were presented in frontal space (red) than in back space (blue) ((OR) = 6.02+/−2.3, z ratio = 4.7, P < 0.001).

Comparing groups within longitudinal spaces, in the frontal space (see red points of Fig. 2), blind people (on the left of Fig. 2) performed worse than sighted people (on the right), showing a lower probability (0.53 vs 0.72) to correctly locate sound delivered in the frontal space ((OR) = 0.41+/−0.13, z ratio = −2.6, P = 0.04). In the back space (see blue points of Fig. 2) the blind group (rhombus) showed no significant differences in accuracy compared to the sighted group (circle), when comparing the probability (0.60 vs 0.30) to correctly locate sound delivered in the back space (OR) = 3.42+/−1.74, z ratio = 2.42, P = 0.07.

**Figure 3:**
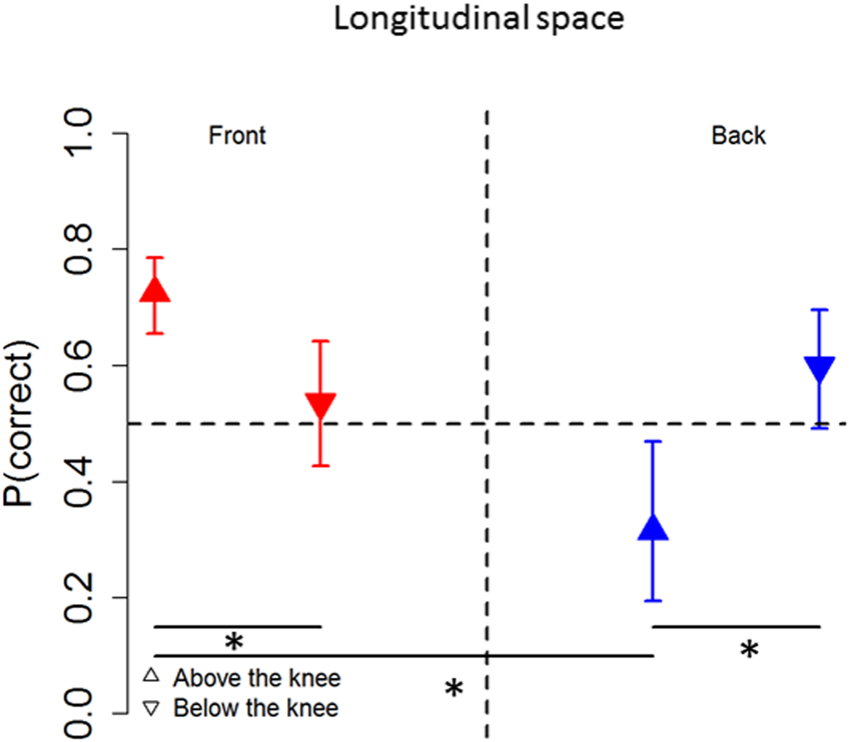
Auditory space around the legs: Interaction between speaker vertical position and longitudinal plane. As can be seen, subjects were more accurate in the back space (blue arrow) when sounds were delivered below the knee (right panel), while above the knee (left panel) subjects performed better in the frontal space (red arrow), (horizontal black bars represent significant differences). Symbols represent LS mean, while errors bars indicate the 95% confidence interval estimated by the LS means.

Figure 3 shows the interaction between longitudinal position and speaker vertical position (X^2^_(1)_ = 82.44, P < 0.001). In frontal space (on the left of Fig. 3), the probability to correctly locate sounds was lower when the sound was delivered below the knee (down arrow) than above the knee (up arrow), ((OR) = 2.28+/−0.47, z ratio = 3.98, P < 0.001). Instead, in the back space (on the right of Fig. 3), the opposite pattern of results was found ((OR) = 0.31+/−0.06, z ratio = −5.24, P < 0.001). These results are common in both groups.

In summary, the results show that in the frontal space, the sighted group localized sounds significantly more accurately than the blind group. While, in the back space, no significant differences were observed between the sighted and blind groups. Moreover, our results showed that participants displayed better localization in the frontal space for sounds delivered above the knee compared with sounds delivered below the knee, while an opposite pattern was found in the back space: better localization was found for sounds delivered below the knee than above the knee. This pattern of results was observed for both groups.

#### Analysis of the probability on perception (i.e. of reporting a sound in frontal space)

To understand if the presence of vision may produce perceptive bias (i.e. a tendency to report a sound in a certain position, independently from where it was delivered), we performed an analysis taking into account the probability to report a sound in frontal space. Analysis of deviance on perceived sounds showed that the presence of vision produce a general bias towards the frontal space. Importantly, this bias is not specific for a portion of space, in other words, it does not interact with any other factors.

Importantly, no significant differences between groups and longitudinal dimension (X^2^_(1)_ = 0.34, P = 0.55), groups and elevation (X^2^_(1)_ = 0.0001, P = 0.99) and between groups, longitudinal position and speaker vertical position (X^2^_(1)_ = 0.008, P = 0.92) were observed.

**Figure 4:**
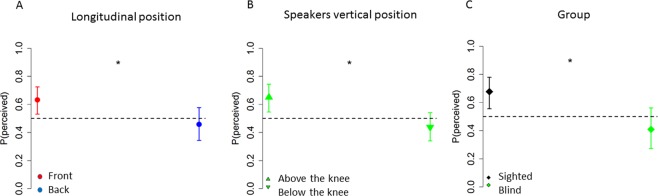
Influence of vision (perceived): Main effect of longitudinal plane (**A**). In the frontal space (red point) subjects reported a significantly higher percentage to perceive the sound as coming from the frontal space when the sound was delivered in the frontal space, compared to when the sound was delivered from the back (blue points). Main effect of elevation (**B**): subject reported a significantly higher percentage to perceive the sound as coming from the frontal space when the sound was delivered above the knee (upper arrow), than below the knee (lower arrow). Main effect of group (**C**): sighted group (black symbol) reported a significantly higher percentage to perceive the sound as coming from the frontal space than blind people (green symbol).

Indeed, we found a main effect of longitudinal position (X^2^_(1)_ = 12.43, P < 0.001): subjects showed a higher probability to report a sound in the frontal than in the back space ((OR) = 1.83+/−0.18, z ratio = 5.88, P < 0.001), see Fig. 4A.

As well, we found a main effect of speaker vertical position (X^2^_(1)_ = 38.89, P < 0.001): the probability to perceive sounds as coming from the frontal space was higher above the knee (0.66 vs 0.44) ((OR) = 2.46+/−0.25, z ratio = 8.76, P < 0.001), see Fig. 4B. Finally, we found a main effect of group (X^2^_(1)_ = 7.1, P = 0.007): sighted people showed a generalized higher probability to report a sound in the frontal space (0.67 vs 0.42) ((OR) = 0.35+/−0.13, z ratio = 2.62, P = 0.0018) than blind people, see Fig. 4C.

In summary, an analysis of the probability of perception (i.e. of reporting a sound as originating in frontal space) showed that sighted people tend to report sounds more frequently in the frontal space, independently of their real position. Interestingly, this does not appear in blind participants, suggesting an important role of vision in the frontal space. However, the bias to report sound in the front did not interact with any specific portion of space.

Finally, we investigated whether the different history of blindness of participants within the blind group may have affected their performance. We applied GLMM with a logit link function and a binomial distribution. With this analysis, a model was fitted for blind participants, taking into account the individual variability of their responses. We regressed, in each trial, the answers of each subject (1 = correct/front, 0 = incorrect/back), as a function of speaker vertical position (above the knee vs below the knee) and longitudinal position (front vs back space), and residual vision (present vs not present), as factors within subjects. We did not find any significant effect which could suggest an influence of residual vision on results.

Then, we applied GLMM with a logit link function and a binomial distribution. With this analysis, a model was fitted for all participants, taking into account the individual variability, blindness onset (age of complete blindness) and blindness duration (length of blindness) in the responses. We regressed, in each trial, the answers of each subject (1 = correct/front, 0 = incorrect/back), as a function of speaker vertical position (above the knee vs below the knee), longitudinal position (front vs back space), and group (blind vs sighted).

The model of correct responses showed that the later the onset of blindness, the greater the probability of correct responses given in the frontal space only (P = 0.03), suggesting that during development vision calibrated hearing in the frontal space. Blind participants with a longer duration of blindness were more correct in the frontal space (P = 0.02), and reported more sounds in the front (P = 0.0008), suggesting that also movement may have a role in spatial representation^55^. If so, it could be expected that motor influences should be more visible in people with longer experience of movement without vision (longer blindness duration) as movement has had more time to calibrate hearing. Blindness onset (P = 0.2) did not affect performance on speaker vertical position, while better performance in the space below the knee is showed by people with a longer duration of blindness (P = 0.02), suggesting that the footstep noise and cane feedback maybe important to calibrate this space.

#### Analysis using percentages

On the same dataset, we implemented two further analyses taking into account the percentage of correct responses and the percentage of responses “Frontal”. On these two parameters we performed a repeated measure ANOVA with factors longitudinal position and speaker vertical position within subjects, and with groups as a between subjects factor. Significant results were further analyzed by post hoc t-test. We considered P < 0.05 as a significant result.

Before starting the analysis, we checked that speaker positions (specific speaker i.e. 1,2,3,4, vs 5.6.7) were similar perceived inside speaker vertical position (above vs below the knee). ANOVA on percentage of correct responses, performed on speakers above the knee, with factors longitudinal position (front vs back) and speaker position (4, 5, 6, 7) within subjects and group as a factor between subjects, showed no interaction between speaker position and the other factors: longitudinal position (P = 0.6), group (P = 0.9) and group and longitudinal position (P = 0.8). The same analysis performed for data below the knee showed no interaction between speaker position and longitudinal position (P = 0.2), group (P = 0.8) and group and longitudinal position (P = 0.7).

#### Analysis of correct answers

**Figure 5:**
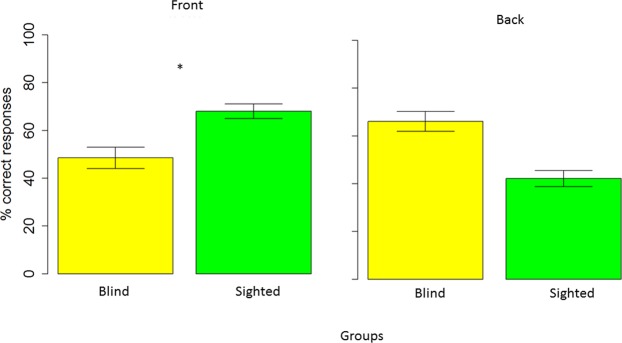
Influence of visual experience (percentage): Interaction between group and longitudinal plane. In the frontal space (left plot), sighted subjects (green bar) reported a significantly higher probability to correctly locate sounds than blind (yellow bar) subjects. In the back space (right plot), no differences were found between groups.

Analysis of variance on correct answers showed an interaction between longitudinal position and group (F _(1, 22)_ = 6.95, P = 0.01). Figure 5 shows this interaction. Comparing groups within longitudinal spaces, in the frontal space (left panel), blind subjects (in yellow in the left panel) reported a significantly lower probability than sighted subjects (in green in the left panel) to correctly locate sounds, (52.14+/−6.6 vs 69.89+/−5.08), t _(22)_ = −2.16, P = 0.04.

Instead, in the back space (see right panel of Fig. 5 no significant difference between the blind group (yellow) and sighted group (green) was observed, t _(22)_ = 1.94, P = 0.06.

**Figure 6:**
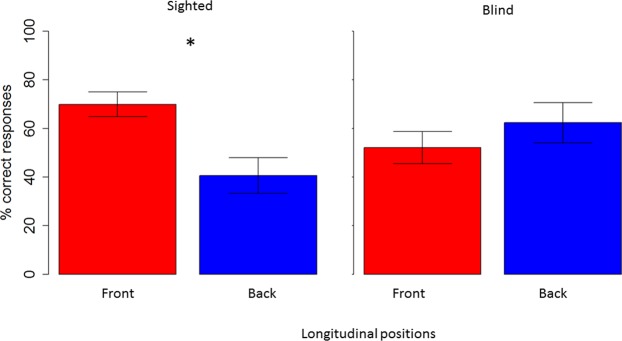
Influence of visual experience (percentage): Interaction between group and longitudinal plane. Blind subjects (right panel) performed similarly in the front (red) and back (blue) space, while sighted subjects (left panel) performed better in the frontal compared to the back space.

Comparing longitudinal spaces within groups, sighted participants (see left panel of Fig. 6 showed higher accuracy in localizing sounds presented in frontal (red) than in the back (blue) space t_(26)_ = 3.27, P = 0.005. Blind participants (see right panel) performed similarly in the two spaces, t _(18)_ = −0.96, P = 0.6.

**Figure 7:**
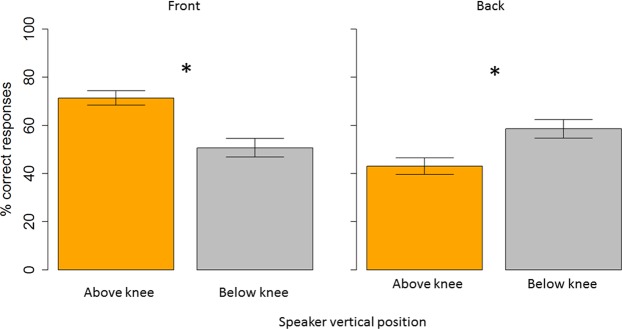
Auditory space around the legs (percentage): Interaction between speaker vertical position and longitudinal plane. In the frontal space (left panel), subjects were more accurate above the knee (orange bar) than below the knee (grey bar). The opposite pattern of results was found in the back space (right panel), where subjects were more accurate at locating sound below the knee.

Figure 7 shows the interaction between longitudinal position and speaker vertical position (F _(1,22)_ = 20.82, P < 0.001). In the frontal space (left panel), accuracy to locate sounds was lower when the sound was delivered below the knee (gray bar) than above the knee (orange bar), t _(46)_ = −2.73, P = 0.004). Instead, in the back space (right panel), the opposite pattern was found, t _(46)_ = 1.79, P = 0.03).

In summary, both analyses (GLMM and percentage) show that in frontal space, the sighted group localized sounds significantly more accurately than the blind group. In back space, no significant differences were observed between the sighted and blind groups. Moreover, our results showed better localization in the frontal space, compared to the back, for sounds delivered above the knee, while better localization in the back occurred when sounds were delivered below the knee. This pattern of results is true for both groups.

#### Analysis of the probability on perception (i.e. of reporting a sound in frontal space)

Interestingly, also with this kind of analysis, we found no significant differences between groups and longitudinal dimension (F_(1, 22)_ = 0.09, P = 0.75), groups and elevation (F_(1, 22)_ = 0.2, P = 0.65) and between groups, longitudinal position and speaker vertical position (F_(1, 22)_ = 0.01, P = 0.91). Indeed, we found a main effect of group (F_(1, 22)_ = 6,95, P = 0.01): sighted subjects showed a higher percentage to report a sound in the frontal space than the blind group t_(46)_ = 2.81, P = 0.007). We also found a main effect of speaker vertical position (F (_1, 22)_ = 20.82, P = 0.001): probability to perceive sounds as coming from the frontal space was higher above the knee t_(47)_ = 3.11, P = 0.002).

In summary, analysis of the probability of perception (i.e. of reporting a sound as originating in frontal space) showed that the sighted group have a generalized bias toward the front. This result is coherent with results found with the GLMM analysis supporting the pivotal role of vision in shaping spatial representation. Indeed, sighted people tend to report stimuli in the frontal space where the presence of vision leads to greater accuracy in localizing sound.

Finally, we investigated, with percentage analysis, whether the different history of blindness of participants within the blind group may have affected their performance. We performed an ANOVA. With this analysis, a model was fitted for blind participants, taking into account the speaker vertical position (above the knee vs below the knee) and longitudinal position (front vs back space), and residual vision (present vs not present), as factors within subjects. We did not find any significant effect which could suggest an influence of residual vision on results.

Next, considering all participants, we took into account the blindness onset (age of complete blindness) and blindness duration (length of blindness), speaker vertical position (above the knee vs below the knee), longitudinal position (front vs back space), and group (blind vs sighted) and performed an ANCOVA (with blindness duration and blindness onset as covariate). Again, we did not find any significant effect which could suggest an influence of residual vision on results.

## Discussion

We investigated the role of prior visual experience on audio spatial perception around the lower body. This study reports, for the first time, the accuracy of auditory localization around the legs using a front-back discrimination task to investigate: i) whether prior visual experience affected performance,

ii) whether performance was better for accurately localizing sounds presented in frontal vs. back space above and below the knee.

Different auditory spatial regions were thus evaluated in sighted and blind individuals. The main findings were:

1. The sighted group performed better than the blind group in frontal space. No significant differences in localization performance were found in back space between the sighted and blind groups.
2. Blind participants performed similarly in the two spaces.
3. Sighted participants showed significantly better performance in the front compared to the back space.
4. In frontal space, performance was higher for sounds presented above the knee than below the knee. In back space, the opposite result was found, performance was better for sounds presented below the knee than above the knee. This result was present in both groups.

Consistent with the experimental hypothesis, the findings showed that blind people performed a front-back discrimination task similarly in the two spaces around the legs. This result can be explained by the lack of visual calibration of the auditory system. Indeed the absence of vision makes perceptually the front space similar to the back space, where the lack of vision produces a degradation of spatial cognition. This result is supported by the result found in the back, where, due to the absence of visual information, the auditory system cannot be calibrated in terms of spatial representations, and so, similar performance was expected between sighted and blind people.

The findings of this study fit in the context of a theoretical framework proposing that the senses play different roles in contributing to the internal representation of various regions of auditory space. Space around the legs is particularly interesting as in this area, while walking, we produce sounds linked to movement. It has been shown that audio motor training can help to improve spatial representation^47,56^. In the current study, we tried to understand if the natural audio-motor training provided by walking could influence the representation of this space. Many works have investigated auditory spatial localization around the upper body, showing better performance of sighted participants compared with blind participants^4,57^. For a review, please see^23^. However, less is known about the influence of the visual modality on other spaces around the body^17,47^, and no previous studies that we are aware of have investigated the effect of visual loss on the auditory representations of space around the legs. Our results showed that both groups were more accurate in localizing frontal sounds when delivered above the knee than below the knee. For sounds originating from the back, the opposite pattern of results was observed, that both groups were more accurate in localizing sounds below the knee than above the knee. The finding that both groups performed similarly suggests that calibration of vertical auditory space (above vs. below the knee) is independent of visual information. Previous studies showed that space around the body is split into several regions, around different body parts^13,15^. Our results can be explained by this model. The different pattern of results for the front (where vision calibrates audition in spatial task) and back spaces (where visual calibration is not possible) suggest that the representation of frontal and back spaces around the legs can be split into at least two regions, above the knee and below the knee. Evidence from a previous study showed that for sighted people, audiomotor training increased the accuracy of front-back discriminations above the knee only, consistent with the current findings that the representation of spaces around the legs can be divided into two regions^47^. In the current study, the different pattern of results for frontal vs. back presented sounds may be due to differences in the sensory feedback used to calibrate auditory space in these regions. Above the knee, hand movements or audiomotor information^47^ may play a role in calibrating space, whereas below the knee, auditory and/or tactile information from footsteps may play a role. However, further study is needed to investigate these possibilities.

The different performance in the four spaces investigated (front vs back, below vs above the knee) suggests that these spaces are differently influenced by sensory experience. Indeed, we found that vision is necessary to accurately represent auditory frontal space, as suggested by the higher performance of the sighted group compared to the blind group in localizing sounds originating in frontal space. In the back, where visual information is unavailable, similar performance between the sighted and blind groups was observed. These results could be influenced by a bias toward the frontal space. Interestingly, only sighted people showed this bias, that it is not confined to a specific part of the frontal space. This finding suggests that the presence of vision emphasizes the frontal space with respect to the back. The conclusion that frontal and back spaces are differently affected by previous visual experience is in line with previous studies showing better performance of blind people compared to sighted in the back space for auditory discrimination^31^, self-localization^41^, and frequency discrimination tasks with sounds originating either from frontal or peripheral locations^42^. Here, we demonstrated that frontal and back spaces are differently affected by previous visual experience for space around the legs, using a front-back discrimination task, where sighted participants were found to be more accurate than blind participants in the frontal space.

In conclusion, our findings support the idea that vision plays an important role in developing an accurate perception of the location of auditory stimuli in the frontal space. Moreover, our data suggest a principal role for sound in the back space around the feet. Indeed, both groups performed well in the back at feet level. We think this effect is related to attention and/or sensory integration rules (when walking we received tactile and audio feedback on the foot, while vision is always forward to our feet, this can produce a lack of visual calibration in the front, while movement may calibrate audition in back space). Further studies are needed to clarify this point. The current study shows for the first time that blind individuals are not able to localize sounds delivered in the front and back spaces (front-back discriminations) around the legs, whereas sighted individuals showed higher accuracy in the frontal space compared to the back. Moreover, we showed that in space where vision is not present, as in the back area, blind and sighted subjects perform similarly. Measurements showed that both groups displayed higher localization performance above the knee than below for frontal sounds, and lower localization performance above the knee for sounds presented from the back. These results show that visual information plays an important role in calibrating hearing in frontal space. Furthermore, the results support and extend previous work demonstrating that auditory space can be divided into different regions above and below the knee^47^.
